# Receptor oligomerization and beyond: a case study in bone morphogenetic proteins

**DOI:** 10.1186/1741-7007-7-59

**Published:** 2009-09-07

**Authors:** Kai Heinecke, Axel Seher, Werner Schmitz, Thomas D Mueller, Walter Sebald, Joachim Nickel

**Affiliations:** 1 Physiologische Chemie II, Biozentrum, Universität Würzburg, Würzburg, Germany; 2 Institut für Humangenetik, Biozentrum, Universität Würzburg, Würzburg, Germany; 3 Universitätsklinikum Würzburg, Abteilung für Molekulare Innere Medizin, Würzburg, Germany; 4 Molekulare Pflanzenphysiologie und Biophysik, Julius von Sachs Institut, Universität Würzburg, Würzburg, Germany

## Abstract

**Background:**

Transforming growth factor (TGF)β superfamily members transduce signals by oligomerizing two classes of serine/threonine kinase receptors, termed type I and type II. In contrast to the large number of ligands only seven type I and five type II receptors have been identified in mammals, implicating a prominent promiscuity in ligand-receptor interaction. Since a given ligand can usually interact with more than one receptor of either subtype, differences in binding affinities and specificities are likely important for the generation of distinct ligand-receptor complexes with different signaling properties.

**Results:**

*In vitro *interaction analyses showed two different prototypes of binding kinetics, 'slow on/slow off' and 'fast on/fast off'. Surprisingly, the binding specificity of ligands to the receptors of one subtype is only moderate. As suggested from the dimeric nature of the ligands, binding to immobilized receptors shows avidity due to cooperative binding caused by bivalent ligand-receptor interactions. To compare these *in vitro *observations to the situation *in vivo*, binding studies on whole cells employing homodimeric as well as heterodimeric bone morphogenetic protein 2 (BMP2) mutants were performed. Interestingly, low and high affinity binding sites were identified, as defined by the presence of either one or two BMP receptor (BMPR)-IA receptor chains, respectively. Both sites contribute to different cellular responses in that the high affinity sites allow a rapid transient response at low ligand concentrations whereas the low affinity sites facilitate sustained signaling but higher ligand concentrations are required.

**Conclusion:**

Binding of a ligand to a single high affinity receptor chain functioning as anchoring molecule and providing sufficient complex stability allows the subsequent formation of signaling competent complexes. Another receptor of the same subtype, and up to two receptors of the other subtype, can then be recruited. Thus, the resulting receptor arrangement can principally consist of four different receptors, which is consistent with our interaction analysis showing low ligand-receptor specificity within one subtype class. For BMP2, further complexity is added by the fact that heterooligomeric signaling complexes containing only one type I receptor chain can also be found. This indicates that despite prominent ligand receptor promiscuity a manifold of diverse signals might be generated in this receptor limited system.

## Background

The bone morphogenic proteins (BMPs), growth and differentiation factors (GDFs) and activins belong to the large transforming growth factor (TGF)β superfamily of secreted signaling molecules [1,2]. The more than 30 TGFβ-like proteins identified in vertebrates to date [3,4] play important roles in all stages of embryogenesis [5]. In the adult organism these factors exhibit a broad range of biological effects and control various processes during regeneration and tissue repair such as growth, growth inhibition, differentiation, apoptosis, and secretion [6,7]. Based on their functional and sequence similarities TGFβ members can be divided into several subfamilies: the TGFβs (TGFβ1, β2, and β3), activins (activin A, B, C, E), BMP2s (BMP2, 4), BMP7s (BMP5, 6, 7), GDF5s (GDF5, 6, 7) and others [1,8]. Signal transduction of TGFβ members is mediated by oligomerizing two different types of transmembrane serine/threonine kinase receptor chains termed type I and type II. Five type II receptors and seven type I receptors have been identified in mammals and the broad range of TGFβ ligands suggests a high degree of promiscuity in ligand-receptor interactions [1,9]. On one hand most receptors can bind several different ligands, and on the other hand most ligands can interact with more than one receptor chain of each subtype. Since members of the TGFβ superfamily transduce signals via a heterooligomeric receptor system, differences in binding affinities and specificities might generate a multiplicity of ligand-receptor complexes with different signaling properties, allowing cellular responses that differ in quality and quantity.

Binding specificities and affinities between ligands and receptors have been analyzed on a semiquantitative basis by crosslinking radioactively labeled ligands with receptors that were overexpressed in cells. Two general binding modes have been observed via this technique. One mode, called 'sequential', is characteristic for TGFβs and activins and involves high affinity binding of the ligand to a type II receptor and subsequent low affinity interaction of this complex with a type I receptor [10,11]. Ligands following this binding mode can be directly crosslinked to a type II receptor but crosslinking to a type I receptor is dependent on the type II receptor presence. The second binding mode, called 'cooperative', is characterized by crosslinking to either the type I or type II receptors and has been proposed for BMPs. However, crosslinking efficiency is enhanced if both receptor types are coexpressed [1].

To better understand receptor activation and the mechanism underlying receptor specificity for TGFβ ligands, we determined binding affinities of different BMPs and GDFs to their cognate receptor ectodomains by surface plasmon resonance. One representative member from each of three BMP/GDF subfamilies was chosen in this study. Binding parameters were evaluated in two ways, (1) by immobilizing the receptor ectodomains of the type I and type II receptors activin receptor (ActR)-I, ActR-IB, BMP receptor (BMPR)-IA, BMPR-IB, ActR-II, ActR-IIB, and BMPR-II, and (2) by immobilizing the ligands. These two setups allow us to obtain data on the individual binding affinity as well as the avidity that is inherently linked to the dimeric nature of the ligands. To compare the binding properties of BMP/GDF receptor interaction with related receptor systems, activin A was included in this study. Possible cooperative interactions between the two receptor types were investigated by studying the formation of ternary complexes consisting of the ligand and the ectodomains of both receptor types on the biosensor chip.

The dimeric nature of the ligands suggests that cooperative binding via multiple interactions between ligand and receptors (avidity) should also exist *in vivo*. Furthermore, since certain ligands such as BMP2, BMP4 or GDF5 can interact independently with type I as well as type II receptors [12,13] an inherent complexity of individual ligand-receptor interactions can be expected on cell surfaces. In addition, since the ligands can bind to other cell surface components such as coreceptors (for example, DRAGON, BAMBI) [14,15] or the extracellular matrix (for example, heparin) [16] the analysis of receptor recruitment and activation is further complicated.

To analyze receptor compositions on cell surfaces and their relation to biological function, BMP2 variants were created lacking the heparin binding sites in order to reduce binding to the extracellular matrix (ECM). Additional amino acid exchanges were introduced resulting in homodimeric or heterodimeric ligands with interrupted receptor binding epitopes. Binding of these variants to receptors expressed on whole cells was analyzed by radioligand binding assays and correlated to their biological activities.

## Results

### Expression and purification of receptor ectodomain and ligand proteins

Since the association rate *k*_on _as well as the binding constant *K*_D _determined from the sensorgrams directly depend on knowledge of the exact concentration of the active analyte, homogeneity and functionality of the analyte protein is essential for obtaining reliable data. Ectodomains (ECDs) of the bacterially derived receptors BMPR-IA, BMPR-IB and ActR-IIB were purified to homogeneity by affinity chromatography employing a BMP2 affinity resin. The receptor ECDs that were expressed in insect cells revealed distinct patterns of bands for each in sodium dodecyl sulfate polyacrylamide gel electrophoresis (SDS-PAGE) analysis under non-reducing conditions. Since upon reduction of the disulfides using β-mercaptoethanol each receptor protein appears as a single band with an apparent molecular weight between 15 and 30 kDa, the bands of higher molecular weight most likely represent incorrectly folded multimers linked by disulfide bridges (data not shown). Purification of only monomeric receptor proteins could be achieved since only monomeric ECDs bound to and were recovered from BMP2 affinity columns. The ECDs ActR-I and ActR-IB derived from insect cells could not be purified by affinity chromatography due to their lack of binding to BMP2. Hence, these receptors were purified to homogeneity by trimethylaminoethyl (TMAE) anion exchange chromatography followed by reverse-phase high performance liquid chromatography (RP-HPLC). All isolated proteins exhibit purities >95% (data not shown).

### Biosensor experiments

**Figure 1:**
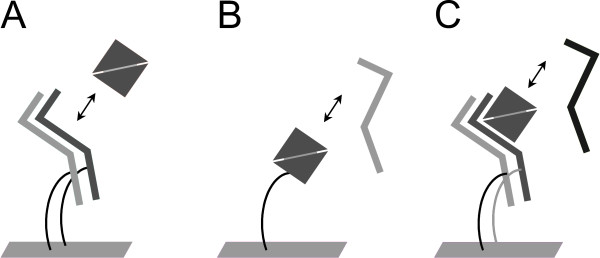
**Experimental layout**. Model of biosensor experiments with ligands as analyte passed over immobilized receptor ectodomains (ECDs) **(a)**, receptor ECDs passed over immobilized ligands **(b) **and ternary complexes formed by perfusing an immobilized type I receptor with the ligand plus the ECD of a type II receptor **(c)**.

As shown by the structures of several ligand-receptor complexes, the dimeric ligands are capable of interacting simultaneously with two receptor molecules of either subtype. Based on this property, the ligands can interact as analyte either with one, or simultaneously with two, immobilized receptors when those are present at sufficient density on the biosensor (Figure 1a). Using the inverse setup, with the ligands immobilized and the receptor as analytes, individual binding of single receptor molecules to the ligands can be determined (Figure 1b). Simultaneous binding of both receptor subtypes to the ligand, as is seen in ternary complex formation, can be recorded using the experimental setup shown in Figure 1c.

### Influence of ionic strength and pH value on binding affinities

The solubility of the BMP ligands strongly depends on pH and ionic strength. In order to find optimal conditions, a series of measurements with varying pH and salt concentration were performed. BMP2 was perfused over biosensor surfaces with the ECDs of BMPR-IA, BMPR-IB or ActR-IIB immobilized and employing different buffers as indicated (Additional file 1).

Similar binding affinities and specificities were observed over a wide range of salt concentrations (150 to 900 mM) and pH conditions (pH 5.0 to 9.5). Thus, the binding of BMP2 to immobilized receptors is unaffected by ionic strength up to 500 mM NaCl. Above 500 mM NaCl the affinities of BMP2 for immobilized receptors decrease up to 10-fold (Additional file 1). As expected, strongest binding is observed at physiological pH. More acidic or basic conditions result in a decrease (3-fold to 10-fold) of the affinities of BMP2 to all tested receptors (Additional file 1).

The observed robustness of binding, independent of pH or ionic strength, can be explained by the nature of the binding interfaces [17-24]. For the interaction of receptors of either subtype with the ligand, the binding is dominated by hydrophobic interactions. Since the association rates are far below the diffusion-controlled limit (<10^7 ^M^-1 ^s^-1^) electrostatic steering seems not to be involved in ligand-receptor interaction.

Based on our results we used 4-(2-hydroxyethyl)-1-piperazineethanesulfonic acid (HEPES) buffer containing 500 mM NaCl at a pH value of 7.4 for all biosensor measurements. Use of this buffer in the interaction analysis yielded binding data which do not differ from those obtained using physiological salt concentrations, but greatly reduced non-specific binding of the ligand to the carboxymethyl cellulose (CM) matrix on the chip surface.

### Binding of ligands to immobilized receptors

Of the ligands tested, the highest binding affinities were observed for BMP2 with preferred binding to the type I receptors BMPR-IA (apparent *K*_D_: 0.8 nM) and BMPR-IB (2.7 nM) and for the GDF5:BMPR-IB interaction (1.3 nM), whereas activin A showed preferential binding to the type II receptor ActR-IIB with similarly high binding affinities (2.1 nM) (see Table 1). In contrast, for BMP7 such a preference in binding to a receptor of either subtype was not detected: comparable affinities were observed instead for the interaction of BMP7 with the type I receptor BMPR-IB (9 nM) and the type II receptors ActR-II (8 nM) and ActR-IIB (9.2 nM). Of the four prototypic ligands tested only BMP7 bound ActR-I, and then very weakly (the sensorgrams could not be evaluated). Since ligand concentrations up to 120 nM were used the apparent *K*_D _value of this interaction is probably larger than 500 nM. The ECD of ActR-IB was not bound by any of the tested ligands.

**Table 1:** Binding parameters of interactions of soluble ligands with immobilized receptor ectodomains (ECDs)

**Ligand (analyte)**	**Type I receptor (immobilized)**						**Type II receptor (immobilized)**					
	**ActR-I**	**ActR-IB**	**BMPR-IA**		**BMPR-IB**		**ActR-II**		**ActR-IIB**		**BMPR-II**	
	
	**Mean**	**Mean**	**Mean**	**SD**	**Mean**	**SD**	**Mean**	**SD**	**Mean**	**SD**	**Mean**	**SD**
	
**BMP-2**												
*k*_on _× 10^-4 ^[M^-1^s^-1^]	NB	NB	50	± 12.1	25	± 4.37	370	± 66.6	280	± 33.6	150	± 25.5
*k*_off _× 10^3 ^[s^-1^]	NB	NB	0.4	± 0.09	0.7	± 0.09	88	± 23.8	18	± 4.68	70	± 19.6
***K***_D _**(kin) [nM]**	**NB**	**NB**	**0.8**	**± 0.37**	**2.7**	**± 0.82**	***14***	***± 6.31***	***6.3***	***± 2.39***	***45***	***± 20.3***
***K***_D _**(eq) [nM]**	**NB**	**NB**	**NE**		**NE**		**24**	**± 1.92**	**9.0**	**± 1.17**	**59**	**± 10.0**

												

**GDF-5**												
*k*_on _× 10^-4 ^[M^-1^s^-1^]	NB	NB	23	± 5.98	39	± 4.68	140	± 15.4	110	± 19.8	110	± 20.9
*k*_off _× 10^3 ^[s^-1^]	NB	NB	4.3	± 0.77	0.5	± 0.16	28	± 4.48	4.5	± 0.59	38	± 8.74
***K***_D _**(kin) [nM]**	**NB**	**NB**	**19**	**± 8.36**	**1.3**	**± 0.56**	***20***	***± 5.40***	***4.0***	***± 1.24***	***36***	***± 15.1***
***K***_D _**(eq) [nM]**	**NB**	**NB**	**NE**		**NE**		**32**	**± 3.84**	**5.6**	**± 0.90**	**46**	**± 8.28**

												

**BMP-7**												
*k*_on _× 10^-4 ^[M^-1^s^-1^]	NE	NB	14	± 2.24	11	± 2.42	120	± 20.4	140	± 30.8	96	± 14.4
*k*_off _× 10^3 ^[s^-1^]	NE	NB	7.9	± 1.19	1.0	± 0.18	6.2	± 0.69	9.0	± 1.26	24	± 6.24
***K***_D _**(kin) [nM]**	> 500*	**NB**	**58**	**± 18.0**	**9.0**	**± 3.87**	***5.1***	***± 1.43***	***6.5***	***± 2.34***	***25***	***± 10.3***
***K***_D _**(eq) [nM]**	> 500*	**NB**	**NE**		**NE**		**8.0**	**± 0.48**	**9.2**	**± 1.28**	**40**	**± 5.20**

												

**Activin-A**												
*k*_on _× 10^-4 ^[M^-1^s^-1^]	NB	NB	NB		NB		130	± 23.4	160	± 22.4	53	± 7.95
*k*_off _× 10^3 ^[s^-1^]	NB	NB	NB		NB		7.5	± 1.65	1.7	± 0.14	29	± 6.96
***K***_D _**(kin) [nM]**	**NB**	**NB**	**NB**		**NB**		***5.7***	***± 2.28***	***1.1***	***± 0.24***	***59***	***± 24.2***
***K***_D _**(eq) [nM]**	**NB**	**NB**	**NB**		**NB**		**6.0**	**± 0.54**	**2.1**	**± 0.15**	**24**	**± 4.08**

The data obtained from measurements with immobilized type I receptor ECDs were fitted to a kinetic model (1:1 Langmuir binding) from which *K*_D _(kin) (bold) is calculated as *k*_off _(× 10^3 ^s^-1^)/*k*_on _(× 10^-4 ^M^-1 ^s^-1^). Due to low but significant binding of BMP7 to AR-I, affinities could not be evaluated exactly but are estimated to be higher than 500 nM (bold, asterisks). The data obtained from ligand binding to immobilized type II receptors were best fitted by equilibrium dose response *K*_D _(eq) (bold). For this interaction the calculation of *K*_D _(kin) (bold, italic) revealed minor differences (<twofold). All data represent mean values of at least three repeated measurements using six different ligand concentrations.

ActR = activin receptor; BMP(R) = bone morphogenic protein (receptor); GDF = growth and differentiation factor; NB = no binding above background detected; NE = could not be evaluated; SD = standard deviation.

The specificity of interactions between the studied receptors and BMP2, BMP7, and GDF5 is only moderate. The receptor BMPR-IA revealed the highest ligand specificity; it binds BMP2 with ≥20-fold higher affinity than GDF5 or BMP7. The interactions of other receptors with these ligands show only discrimination with a 10-fold difference in binding affinity. Among the ligands, GDF5 exhibits the highest receptor specificity, binding preferentially to BMPR-IB and ActR-IIB. The type I receptor specificity of GDF5 is defined by a single residue (Arg57), which is located in the pre-helix loop in the center of the type I receptor binding epitope [25].

For some of our data similar results have been published by other groups [18,26]. However, affinities of other ligand-receptor interactions differ by more than two orders of magnitude. Of note, the affinities of activin A for binding to the immobilized type II receptors ActR-II and ActR-IIB are reported as 10-fold to 100-fold higher compared to our data. The discrepancy is mainly due to lower dissociation rates (*k*_off_) that are reported by Greenwald *et al*. [18,27]. In addition, the affinity of BMP7 for BMPR-IA according to our measurements is 20-fold higher than reported by Allendorph *et al*. [26]. One explanation might be differences in the chip surface density of the immobilized receptor. At low immobilization levels the distances between individual receptors might be too large to allow for simultaneous interaction of the dimeric ligand with two immobilized receptors. By contrast, at very high densities steric hindrances could occur. An investigation into this has been reported for the interaction of activin A with the type II receptor ActR-IIB [27].

**Figure 2:**
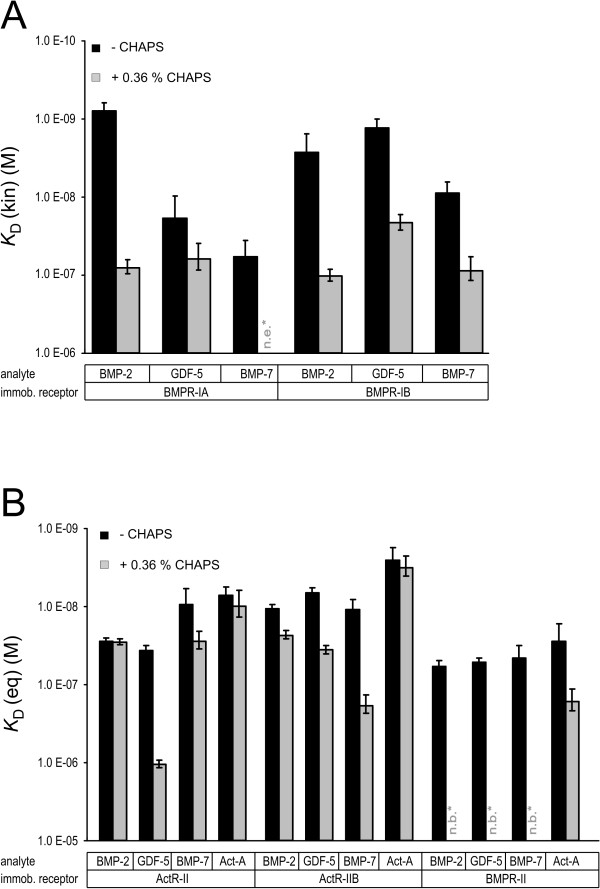
**Influence of 3-[(3-cholamidopropyl)dimethylammonio]-1-propanesulfonate (CHAPS) for ligand-receptor interaction**. Binding affinities of the ligands bone morphogenic protein (BMP)2, BMP7 and growth and differentiation factor (GDF)5 to the immobilized type I receptors BMP receptor (BMPR)-IA and BMPR-IB **(a) **and those of the same ligands plus activin A to the type II receptors activin receptor (ActR)-II, ActR-IIB, and BMPR-II **(b) **are depicted as bar diagrams. The data represent mean values of two individual experiments using six different ligand concentrations. Standard deviations are indicated by error bars.

However, another explanation for the latter discrepancy might be due to the usage of the detergent 3-[(3-cholamidopropyl)dimethylammonio]-1-propanesulfonate (CHAPS). The results obtained from measurements with 0.36% CHAPS added to HBS500 buffer (see Methods) differ in most of the cases, some dramatically, from those obtained without CHAPS (Figure 2). Only the interaction of activin A with ActR-II and ActR-IIB is unaffected, whereas all other ligand-receptor interactions show a reduced affinity. The binding affinity of BMP7 to BMPR-IA was reduced 20-fold. The sensorgrams for the BMP7:BMPR-IA (Additional file 2) interaction could not be directly evaluated, but a correlation of the resonance units obtained with an 80 nM ligand solution and the known *R*_max _value of the sensor chip yields an estimation of the apparent *K*_D _value of approximately 2 μM. However, not only was the interaction between ligands and type I receptors changed in the presence of the detergent CHAPS, but binding specificity to the type II receptors was also altered. Whereas binding of GDF5 to ActR-II showed only a 30-fold decrease, binding of the ligands to BMPR-II was completely abolished in the presence of CHAPS. Thus, the presence of CHAPS not only alters the binding affinities but also influences ligand-receptor specificities in the majority of the interactions investigated here.

### Binding of receptors to immobilized ligands

Due to the measurement of the 1:1 interaction and hence the lack of avidity, apparent affinities are much lower when the setup is based on immobilized ligands and using the soluble receptor ectodomains as analytes (Table 2). Binding constants range from 48 nM for the interaction of BMPR-IA with BMP2 up to 60 μM for the binding of BMPR-II to immobilized GDF5. The very weak affinities of the 1:1 interaction of BMPR-II to BMP2, BMP7, and GDF5 have been recently reported by Yin *et al*. [28]. Under this setup again BMPR-IA shows the strongest overall binding (among the type I receptors) to BMP2 (*K*_D_: 48 nM). A similar value was reported by Sachse *et al*. [29] for this interaction and for the binding of BMPR-IA to BMP4 [30,31], which is plausible considering that the type I receptor binding epitope (wrist epitope) of BMP2 and BMP4 share 100% amino acid identity [32]. Interestingly, using this setup with immobilized ligands, the type I receptor ActR-I measurably interacts only with BMP7.

**Table 2:** Binding parameters of interactions of soluble receptors with immobilized ligands.

**Ligand**	**Type I receptor (analyte)**							**Type II receptor (analyte)**					
	**ActR-I**		**ActR-IB**	**BMPR-IA**		**BMPR-IB**		**ActR-II**		**ActR-IIB**		**BMPR-II**	
	
	**Mean**	**SD**	**Mean**	**Mean**	**SD**	**Mean**	**SD**	**Mean**	**SD**	**Mean**	**SD**	**mean**	**SD**
	
**BMP-2**													
*k*_on _× 10^-4 ^[M^-1^s^-1^]	NB		NB	3.9	± 0.63	2.3	± 0.56	NE		NE		NE	
*k*_off _× 10^3 ^[s^-1^]	NB		NB	1.9	± 0.47	8.0	± 1.38	> 100		> 100		> 100	
***K***_D _**(kin) [nM]**	**NB**		**NB**	**48**	**± 19.6**	**350**	**± 146**	***NE***		***NE***		***NE***	
***K***_D _**(eq) [nM]**	**NB**		**NB**	**NE**		**NE**		**3800**	**± 608**	**3100**	**± 527**	**13000**	**± 2470**

													

**GDF-5**													
*k*_on _× 10^-4 ^[M^-1^s^-1^]	NB		NB	0.5	± 0.162	0.3	± 0.09	NE		NE		NE	
*k*_off _× 10^3 ^[s^-1^]	NB		NB	17	± 1.90	1.0	± 0.124	> 100		> 100		> 100	
***K***_D _**(kin) [nM]**	**NB**		**NB**	**3300**	**± 1439**	**300**	**± 123**	***NE***		***NE***		***NE***	
***K***_D _**(eq) [nM]**	**NB**		**NB**	**NE**		**NE**		**22000**	**± 2420**	**4700**	**± 658**	**60000**	**± 9600**

													

**BMP-7**													
*k*_on _× 10^-4 ^[M^-1^s^-1^]	NE		NB	0.3	± 0.05	3.1	± 0.38	NE		NE		NE	
*k*_off _× 10^3 ^[s^-1^]	NE		NB	5.4	± 0.70	23	± 5.84	> 100		> 100		> 100	
***K***_D _**(kin) [nM]**	**NE**		**NB**	**1900**	**± 589**	**750**	**± 283**	***NE***		***NE***		***NE***	
***K***_D _**(eq) [nM]**	**58000**	± 29140	**NB**	**NE**		**NE**		**880**	**± 61.6**	**2500**	**± 300**	**9100**	**± 1365**

													

**Activin-A**													
*k*_on _× 10^-4 ^[M^-1^s^-1^]	NB		NB	NB		NB		30	± 3.30	14	± 2.38	10	± 1.23
*k*_off _× 10^3 ^[s^-1^]	NB		NB	NB		NB		44	± 10.6	9.6	± 2.78	76	± 19.3
***K***_D _**(kin) [nM]**	**NB**		**NB**	**NB**		**NB**		***180***	**± 63.0**	***88***	**± 40.5**	***890***	**± 335**
***K***_D _**(eq) [nM]**	**NB**		**NB**	**NB**		**NB**		**NE**		**NE**		**NE**	

The data obtained from the interaction soluble type I receptor ectodomains (ECDs) with the immobilized ligands were fitted to the 1:1 Langmuir binding model and the *K*_D _(kin) (bold) calculated as *k*_off _(× 10^3 ^s^-1^)/*k*_on _(× 10^-4 ^M^-1 ^s^-1^). Due to the fast kinetics of the interaction between type II receptors as analyte and the immobilized ligands BMP2, BMP7 and GDF5, the data could only be fitted by equilibrium dose response *K*_D _(eq). The dissociation rate constants (*k*_off_) of these interactions are >100 (× 10^3 ^s^-1^). All data represent mean values of three repeated measurements using at least six different analyte concentrations.

ActR = activin receptor; BMP(R) = bone morphogenic protein (receptor); GDF = growth and differentiation factor; NB = no binding above background detected; NE = could not be evaluated; SD = standard deviation.

Regarding the ligand specificities of the receptors, the results are similar to those observed with the reciprocal setup using immobilized receptors. Owing to the lack of avidity all affinities are 'scaled' down by a factor of 50 to 1,000. However, for BMP2 and GDF5 the binding to the type II receptors benefits much more from avidity effects compared to type I receptor binding. For BMP7, which binds type I and type II receptors with similar affinities, no such significant receptor subtype specific effect on the avidity is observed. In the case of activin A simultaneous binding of the ligand to 2 type II receptors also leads to an increased affinity by a factor of 30 to 40, direct binding of activin A to type I receptors is not observed independent of the biosensor setup. The lack of type I receptor binding of activin A can be possibly explained by the known structures of activin A:ActR-II complexes, which show that the type I receptor epitope in activin A might be structurally disrupted in the absence of the type II receptors [23,27].

### Binding affinities in ternary complexes

The crystal structures of the BMP2:BMPR-IA:ActR-II [17] and BMP2:BMPR-IA:ActR-IIB [24] ternary complexes clearly demonstrate the lack of any receptor:receptor contacts. Furthermore, no gross conformational changes are observed in the ligand dimer architecture of BMP2 upon complex formation, in contrast to activin A and TGFβ3. Consequently, a cooperative recruitment of the type II receptor ectodomains could be excluded from Biacore measurements [24]. To determine whether all type II receptor ectodomains bind to BMP2, BMP7 and GDF5 with identical affinities independent of the presence of a type I receptor, ternary complexes were generated on the biosensor matrix as described in the Methods section (Figure 1c, Table 3). The results of the 'ternary' interactions reveal only marginal differences compared to those obtained for individual receptor-ligand interactions (see Tables 2 and 3). All differences, except for the interaction of ActR-IIB with the BMP7:BMPR-IB_immobilized _complex, are within a factor of two and thus not significant considering the standard deviations of regular biosensor measurements. An increase in affinities due to cooperativity, as shown for the binding of BMP7 to ActR-I in the presence of ActR-II [18], could not be detected in our experiments. The detection of ternary complex formation via the immobilized type I receptor ActR-I was not possible due to its low ligand binding capabilities. The reverse detection to measure the binding of soluble type I receptor ECDs to a preformed ligand:type II receptor complex with the type II receptor serving as the anchor to the biosensor could not be performed, since the fast dissociation rates *k*_off _for ligand type II receptor interaction impeded a coinjection setup, which is the experimental basis for these measurements.

**Table 3:** Binding affinities of soluble type II receptors in ternary complexes

**Type I receptor (immobilized)**	**Ligand (analyte I)**		**Type II receptor (analyte 2)**		
			
			**ActR-II**	**ActR-IIB**	**BMPR-II**
BMPR-IA	BMP2	*K*_D _(eq), nM	4,200	2,800	22,000
BMPR-IB		SD	± 714	± 357	± 6,160
BMPR-IA	GDF5	*K*_D _(eq), nM	20,000	2,900	32,000
BMPR-IB		SD	± 3,660	± 339	± 5,632
BMPR-IA	BMP7	*K*_D _(eq), nM	1,500	7,000	16,000
BMPR-IB		SD	± 130	± 1,015	± 2,592

The data obtained from binding of type II receptor ectodomains (ECDs) as analyte to the preformed complexes were fitted by equilibrium dose response *K*_D _(eq). The data represent mean values of two measurements using at least six different type II receptor concentrations.

ActR = activin receptor; BMP(R) = bone morphogenic protein (receptor); GDF = growth and differentiation factor; SD = standard deviation.

In summary, our data clearly indicate an independent binding of the ectodomains of type I and type II receptor to the ligands BMP2, BMP7, and GDF5. However, since in surface plasmon resonance (SPR) measurements only isolated extracellular domains of the receptors are used, the cooperative recruitment of the type II receptor chains that are observed in crosslinking experiments on cells must therefore be generated by an alternative mechanism, such as the interaction of transmembrane or intracellular domains of the receptors.

### Different types of binding kinetics

Generally, two types of binding kinetics could be observed in our experiments. The first type, which is observed for the interaction of BMP2, BMP7, and GDF5 with the immobilized type I receptors BMPR-IA and BMPR-IB, can be considered 'slow' being characterized by relatively slow association *k*_on _(1 to 5 × 10^5 ^M^-1 ^s^-1^) and dissociation rates *k*_off _(0.4 to 8 × 10^-3 ^s^-1^) (see Additional file 3). The second type, which is seen for the majority of BMP2, BMP7 and GDF5 type II receptor interactions, is 'fast' exhibiting fast association *k*_on _(>10^6 ^M^-1 ^s^-1^) and dissociation rates *k*_off _(>10^-2 ^s^-1^) (see Additional file 3). The sensorgrams measuring ternary complex formation clearly display both types of binding kinetics, the slow association and dissociation of the ligand to/from the immobilized type I receptor ectodomain and the fast binding kinetics for the interaction of the soluble type II receptor ectodomain with the preformed complex (Additional file 3).

**Figure 3:**
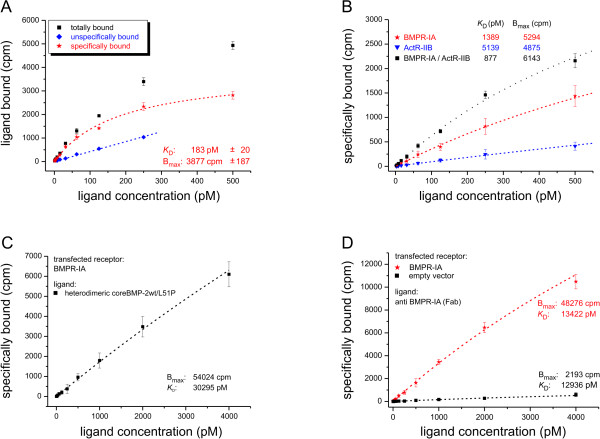
**Binding of radiolabeled proteins on cell surfaces**. **(a) **Dose-dependent binding of iodinated core bone morphogenic protein (wild type) (coreBMP2wt) to C2C12 cells (total binding, black squares). Unspecific binding as determined by addition of a 1,000-fold excess of cold ligand (blue diamonds) was subtracted resulting in specific binding of the ligand (red stars). **(b) **Comparison of specific binding of coreBMP2wt to COS-7 cells transfected with either BMP receptor (BMPR)-IA or activin receptor (ActR)-IIB or cotransfected with both receptors chains. **(c) **Specific binding of the iodinated heterodimeric coreBMP2/L51P mutant to BMPR-IA transfected COS-7 cells using ligand concentration up to 4 nM. **(d) **Specific binding of a radiolabeled anti-BMPR-IA Fab fragment to either untransfected (black squares) or BMPR-IA transfected (red asterisks) COS-7 cells. At all cases specific binding was fitted to a one-site binding model resulting in the indicated values for *K*_D _and *B*_max_.

The 1:1 interactions of the soluble type I and type II receptor ectodomains to the immobilized ligands show principally comparable characteristics (in terms of fast and slow) to those of the 1:2 interactions, which are observed in the inverse situation (compare figures in Additional file 3). Binding kinetics of the 1:1 interaction are generally characterized by faster dissociation rates *k*_off_. This is expected since on the biosensor with the ligand being immobilized, the binding epitopes of the ligand act independently, thus a dissociation of the receptor analyte is irrevocable. In the 1:2 interaction dissociation of the ligand analyte from one receptor does not automatically cause the release of the ligand from the biosensor. Since the ligand is still coupled via the second receptor, fast rebinding can occur and hence the dissociation is dramatically decreased. Noteworthy is the very fast dissociation of the type II receptor analytes from the immobilized BMP2, BMP7, and GDF5 resulting in sensorgrams with an almost rectangular shape (Figure 3b). Since data acquisition can only proceed with a limited sampling frequency (2.5 Hz) an evaluation of the kinetic rate constants is not feasible. Thus, the dissociation rates *k*_off _can be estimated to be certainly >10^-1 ^s^-1 ^but more precise analysis cannot be provided here. Hence, no predictions with regard to the association rates can be made.

The lifetimes of individual ligand-receptor complexes can be deduced from the dissociation rates. For the 1:2 interaction of BMP2, BMP7 and GDF5 with the type I receptors BMPR-IA and BMPR-IB rather long complex lifetimes (t_1/2 _= (ln2)/*k*_off_) on the order of 2 to 30 min can be calculated, whereas ligand:type II receptor complexes with the type II receptors anchored to the sensor surface exhibit half-lives of the order of a few seconds (1 to 15 s). For the 1:1 interaction, which can be considered the initial binding event in the case of a sequential binding mechanism, complex lifetimes are significantly reduced. However, the lifetimes of almost all BMP2, BMP7, and GDF5 type I receptor (1:1) complexes are still longer than those determined for the 1:2 interactions of these ligands with the type II receptors. Only activin A can form complexes with type II receptors that exhibit half-lives longer than 1 min.

Our data strongly suggest that, in all ligand-receptor systems tested here, one defined receptor subtype serves as an anchor for the recruitment of the ligand from the supernatant to the membrane surface. The other receptor subtype either does not interact with the ligand (that is, activin A with ActR-IB) or binds with a fast binding kinetic as observed for the BMP2 or GDF5 type II receptor complexes and thus cannot efficiently act as a membrane anchor. These data consequently suggest a sequential binding mode for BMP2 and GDF5, with an initial recruitment via type I receptors and a subsequent binding of the type II receptors to this intermediate ligand:type I receptor complex.

### Ligand binding on whole cells

The presence of four receptor binding epitopes in the dimeric ligand creates the possibility of a whole set of individual ligand-receptor interactions on cell surfaces. In addition the ligands can interact with other cell surface components such as coreceptors (for example, DRAGON, BAMBI) [14,15] or the extracellular matrix (for example, heparin) [16]. In order to lower interactions with the extracellular matrix, we created BMP2 ligands lacking the heparin binding sites (so-called coreBMP2 variants, see Methods section). In biosensor analyses the variant core BMP2 wild type (coreBMP2wt) exhibits receptor binding characteristics identical to those of wtBMP2 indicating that the N-terminal sequences are not involved in receptor interaction. For the homodimeric coreBMP2L51P variant no binding to type I receptors is detected (*K*_D _> 1 μM), in agreement with published data [33]. Binding to type II receptors is identical to that of wtBMP2, confirming that the mutation L51P solely destroys type I receptor binding. In the case of the heterodimeric coreBMP2wt/BMP2L51P variant a binding constant of 50 nM was determined for the interaction with BMPR-IA and of 350 nM for the binding to BMPR-IB. Interestingly, the same binding constants could be determined when either the ligand or the receptors were immobilized. Furthermore, these values resemble the 1:1 interactions of BMPR-IA or BMPR-IB with wtBMP2 (see Table 2). So far no mutations in BMP2 have been found that are able to completely abolish type II receptor binding. The heterodimeric coreBMP2wt/A34D variant binds type II receptor ectodomains (immobilized on the biosensor) with only 3-fold lower binding affinity, and the homodimeric coreBMP2A34D variant with 10-fold lower binding affinity, compared to wtBMP2. Since a real 1:1 ligand:type II receptor interaction cannot be simulated with these ligands they were not suitable for radioligand binding assays.

We analyzed the binding of iodinated coreBMP2wt to C2C12 cells (Figure 3a). When ligand concentrations up to 500 pM were used a binding constant of about 180 pM was determined with roughly 12,000 binding sites calculated per cell. Both values agree with previously published binding data employing other BMP responsive cells [34,35].

Of note, 30% of total binding to C2C12 cells was non-specific even when coreBMP2wt was used. Since nothing is known about the detailed receptor composition for the binding sites detected in these cells similar experiments were carried out using transiently transfected COS-7 cells (Figure 3b). The conditions were chosen to keep the number of binding sites similar to those observed in non-transfected C2C12 cells, however the affinities for the ligands were at least fourfold lower. Importantly, the values observed for the binding of BMP2 to cells transfected with BMPR-IA or ActR-IIB were basically identical to those of the 1:2 interactions determined from Biacore measurements (see Table 1). Cotransfection of both receptor subtypes resulted in a marginal increase in binding affinities (<twofold), similar to what was observed from Biacore measurements when the ectodomains of both receptor subtypes were immobilized simultaneously on the biosensor (data not shown). These data clearly show that also on whole cells only very weak cooperativity, if any, exists in BMP2-mediated receptor recruitment.

**Figure 4:**
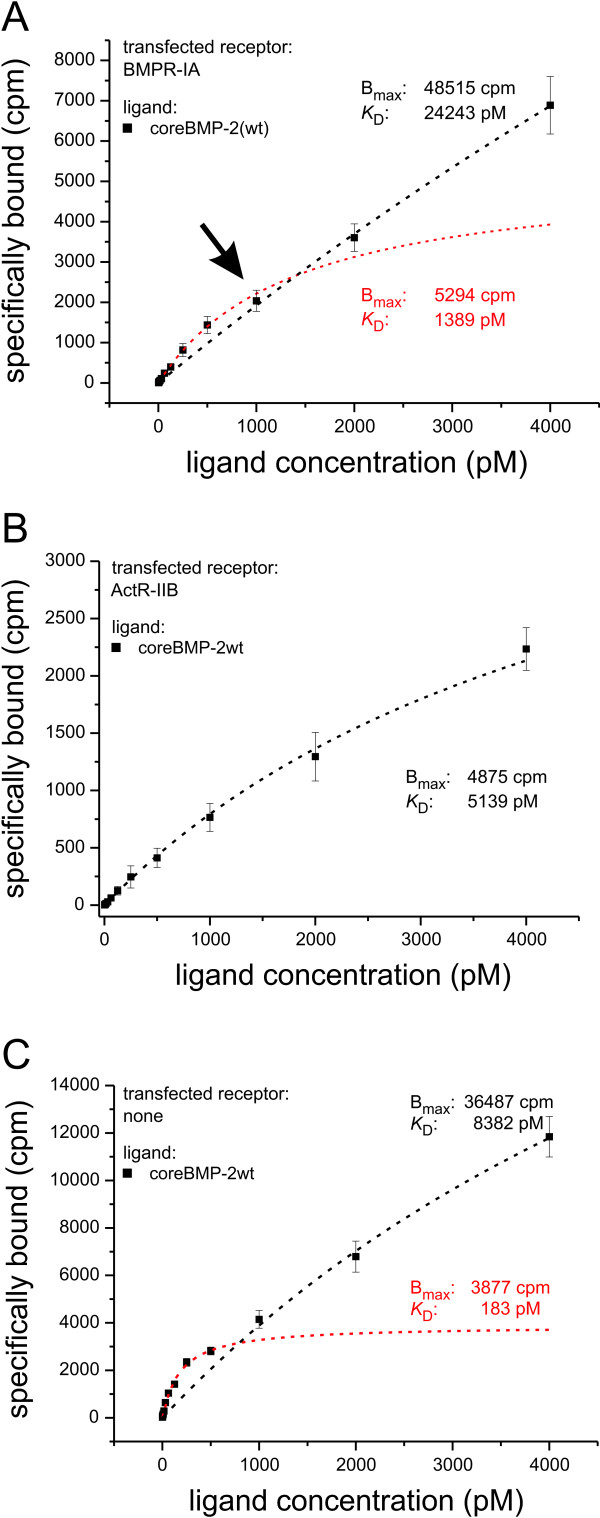
**Binding of radiolabeled ligands using higher ligand concentrations**. **(a) **Specific binding of core bone morphogenic protein (wild type) (coreBMP2wt) to BMP receptor (BMPR)-IA transfected results in a biphasic binding curve with a pronounced break (marked by arrow). Fitting the curve separately (0 to 500 pM and 1,000 to 4,000 pM) to a one-site binding model the indicated values for *K*_D _and *B*_max _were achieved. **(b) **Specific binding of coreBMP2wt to COS-7 cells transfected with activin receptor (ActR)-IIB and **(c) **to untransfected C2C12 cells.

Using up to 500 pM concentrations of the heterodimeric coreBMP2wt/L51P variant the resulting binding curves did not enter the plateau phase and thus could not be fitted to a one-site binding model. With higher ligand concentrations a binding constant *K*_D _of approximately 30 nM was obtained resembling the binding affinity for the 1:1 BMP2:BMPR-IA interaction as determined from Biacore measurements (Figure 3c). Interestingly, the number of binding sites seems about 10-fold higher (approximately 150,000 per cell) compared to the measurements obtained with homodimeric wild-type coreBMP2 (see Figure 3a). Since we cannot exclude that other sites beside the transfected receptor are bound at higher ligand concentrations, the BMPR-IA binding sites were directly determined using a radiolabeled Fab fragment (AbyD, Morphosys, Martinsried, Germany), which binds specifically to the ectodomain of BMPR-IA (Figure 3d). For mock-transfected and BMPR-IA transfected cells an identical binding constant of *K*_D _approximately 13 nM was obtained for the Fab fragment, which is again consistent with Biacore measurements (data not shown). Furthermore, the number of BMPR-IA-derived binding sites as determined from the Fab-fragment binding is basically identical to those found in the measurements using the heterodimeric coreBMP2wt/L51P variant. In mock-transfected cells the number of BMPR-IA-derived binding sites is approximately 25-fold lower. Thus COS-7 cells express only minor amounts of BMPR-IA endogenously and the majority of the signal in the transfected cells is generated from the interaction with the ectopically expressed BMPR-IA. Due to the monovalent nature of our Fab fragment the number of binding sites most likely accounts for individual BMPR-IA molecules on the cell surface. Consequently, the interaction of coreBMP2wt with BMPR-IA should result in similar values for maximal ligand binding (*B*_max_) at higher concentrations. However, when we used higher concentrations of coreBMP2wt we obtained a biphasic binding curve indicating the presence of two different kinds of binding sites (Figure 4a). Separate evaluation of the binding affinities for the lower (0 to 500 pM) and higher (1,000 to 4,000 pM) concentrations yields *K*_D _values of 1.4 and 25 nM resembling the affinities obtained from Biacore experiments for the 1:2 (high affinity) and the 1:1 (low affinity) interaction. Importantly, the majority (90%) of the total binding sites are low affinity sites, which most likely reflect receptor monomers, whereas only 10% of the binding sites exhibit high binding affinity. These sites most likely represent receptors that are arranged as preformed dimers or even in higher ordered structures thereby allowing a simultaneous 1:2 interaction.

Repeating the experiment using ActR-IIB transfected cells to measure the binding of coreBMP2wt at higher concentrations did not produce a biphasic binding curve (Figure 4b). Fitting analysis of the binding data at higher or lower ligand concentration resulted in identical values for *K*_D _and *B*_max_. To determine whether the rather small *B*_max _values are due to weaker expression of ActR-IIB, expression levels were independently tested using fluorophore tagged receptors and western blot analysis of whole cell lysates. Since no significant differences were detected between BMPR-IA and ActR-IIB transfected cells, this suggests that the majority of the ActR-IIB receptors on the cell surface are not occupied by the ligand even at concentrations of 4 nM (data not shown).

To determine, whether non-transfected BMP2 responsive cells exhibit the same distribution of monomeric or dimeric receptor assemblies C2C12 cells were incubated with iodinated coreBMP2wt (Figure 4c). Similar to BMPR-IA transfected COS-7 cells a biphasic binding curve was observed. The number of binding sites at lower and higher concentrations suggest a similar distribution of high and low affinity receptor sites, but binding affinities were four times higher for both 1:1 and 1:2 interactions compared to BMPR-IA transfected COS-7 cells. It remains unclear if the very tight binding in untransfected BMP2 responsive cells is due to the interaction of the ligand with both endogenously expressed type I and type II receptor chains resulting in a heterohexameric complex. The high affinity might likewise due to involvement of affinity-enhancing coreceptors such as DRAGON, a member of the repulsive guidance molecule (RGM) family, which might facilitate ligand binding to dimeric as well as to monomeric receptors. Expression of all three RGM family members could be detected in C2C12 cells by real-time RT-PCR experiments. The highest expression levels found for DRAGON (RGMb) were approximately 20-fold lower compared to those of BMPR-IA (data not shown).

### Biological activity

**Figure 5:**
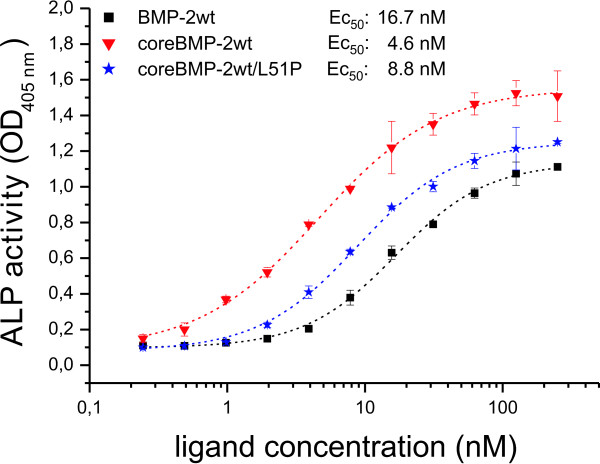
**Biological activity of bone morphogenic protein (BMP)2 variants**. The dose-dependent induction of alkaline phosphatase (ALP) activity in serum starved C2C12 cells is shown for the indicated ligands BMP2 wild type (BMP2wt) (black squares), coreBMP2wt (red triangles) and the heterodimeric coreBMP2wt/L51P variant (blue asterisks). The background absorption at 405 nm of 0.09 ± 0.0075 was not subtracted to indicate the signal to noise ratio.

Our results indicate a similar distribution of monomeric and dimeric receptor arrangements in non-transfected BMP2 responsive cells and in cells transfected with BMP receptors suggesting consequences for downstream signaling events. We therefore used induction of alkaline phosphatase (ALP) expression to monitor the effect of different receptor complex arrangements. In C2C12 cells BMP2 induces ALP activity in a dose-dependent manner requiring about 20 nM BMP2 for half-maximal response [32]. The presence and functional importance of BMPR-IA for this ALP activation has been reported previously [36,37]. Other receptors present in our C2C12 cells are ActR-I, ActR-II and BMPR-II, whereas BMPR-IB and ActR-IIB seem to be expressed at very low levels (data not shown). Since BMP2 cannot efficiently activate cells expressing ActR-I as the only type I receptor, signal transduction in C2C12 cells is most likely mediated via BMPR-IA [38,39]. Interestingly, the concentration for half maximal response for ALP induction in these cells correlates well with the *K*_D _value of the 1:1 BMP2:BMPR-IA interaction, and thus suggests that ALP induction might be controlled via an isolated (not dimeric) type I receptor architecture. Therefore, ALP assays were performed employing the homodimeric coreBMP2L51P and the heterodimeric coreBMP2wt/L51P variants. The homodimeric coreBMP2L51P variant fails to induce ALP expression, which is in agreement with results published earlier using similar BMP variants that have both type I receptor sites destroyed [33,40]. However, the heterodimeric coreBMP2wt/L51P variant shows induction of ALP expression similarly (difference <twofold) as coreBMP2wt (Figure 5). For comparison, wild-type BMP2 and the N-terminal truncated coreBMP2wt variant differ about fourfold in ALP induction, indicating that the presence of heparin binding sites influences the induction of ALP expression more so than does the complete ablation of one type I receptor binding epitope.

### SMAD phosphorylation

In addition to ALP induction we also studied SMAD-1 phosphorylation in C2C12 cells. We performed an initial timecourse analysis of SMAD-1 phosphorylation from 30 min to 2 h after ligand addition [41] and also monitored the influence of the ligand concentration.

**Figure 6:**
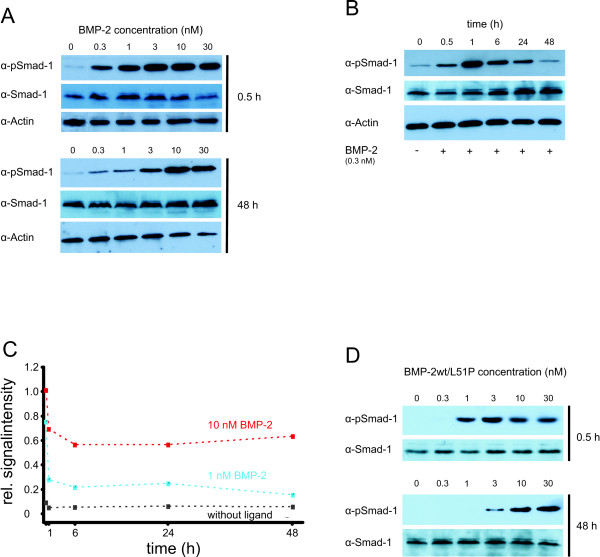
**SMAD phosphorylation**. **(a) **A concentration-dependent phosphorylation of SMAD-1 mediated by bone morphogenic protein (BMP)2 is shown at the indicated timepoints in C2C12 cells by western blotting. Analysis of SMAD-1 and actin levels acts as loading control. **(b) **Time-dependent phosphorylation of SMAD-1 by BMP2 at a concentration of 0.3 nM. **(c) **Diagram of the time-dependent phosphorylation levels of SMAD-1 without ligand or induced by BMP2 at the indicated concentrations. The data were obtained by scans of western blot exposures. pSMAD signals were quantified and normalized to total SMAD-1 levels using the software ImageJ. **(d) **Concentration-dependent phosphorylation of SMAD-1 mediated by heterodimeric core BMP wild type (coreBMP2wt)/L51P.

After incubating the cells with coreBMP2wt for 30 min half-maximal phosphorylated SMAD (pSMAD) levels were obtained using approximately 300 pM of ligand (Figure 6a). We then extended the time course analysis to examine the kinetics of long-term stimulation. Interestingly, the ligand concentration required for SMAD phosphorylation increases significantly with the time of ligand exposure (Figure 6b). After 48-h incubation 10-fold to 30-fold higher ligand concentrations compared to short-term incubation (a 1-h period) were necessary to induce half-maximal pSMAD levels (see Figure 6a, c). At ligand concentrations >3 nM high pSMAD-1 levels could also be observed after 6 h and 24 h of ligand exposure indicating a permanent activation of the SMAD pathway (data not shown). Interestingly, the total SMAD-1 protein levels also marginally increased over time, but ligand independently. Dose-dependent phosphorylation of SMAD-1 could be also observed upon stimulation with the heterodimeric coreBMP2wt/L51P variant. Similarly, sensitivity to ligand exposure decreased over time although not to the extent observed with wild-type ligand (Figure 6d). Importantly, after 48 h of ligand exposure, half-maximal pSMAD-1 levels were achieved using the same concentration of the heterodimeric BMP2 variant (approximately 3 nM) as observed for wtBMP2. This is consistent with the comparable ability of wtBMP2 and heteromeric wtBMP2/L51P variant to induce ALP, in that at variant concentrations required for half-maximal ALP induction (10 to 20 nM) pSMAD-1 levels are similarly high.

### Inhibition of the SMAD and mitogen-activated protein (MAP) kinase pathway

The important points to consider are whether SMAD phosphorylation and induction of ALP expression are coupled through a common signaling cascade. It is supposed that SMAD phosphorylation leads to differentiation of C2C12 cells into the osteoblastic lineage, but it is unclear whether SMAD phosphorylation is also required for the induction of ALP gene expression at later timepoints. Recently, a small-molecule inhibitor of BMP signaling, called dorsomorphin, was demonstrated to perturb dorsoventral axis formation in Zebrafish [42]. This substance selectively inhibits BMP type I receptors ActR-I, BMPR-IA and BMPR-IB and thereby prevents phosphorylation of SMAD1/5/8 proteins. SMAD2/3 phosphorylation as well as phosphorylation of the p38 MAP kinase is not affected by dorsomorphin. The p38 MAP kinase pathway contributes to chondrogenesis induced by GDF5 in ATDC-5 cells as well as to osteogenic differentiation of C2C12 cells mediated by BMP2 [43,44]. Several small molecule inhibitors of p38 MAP kinase activation are currently available, however not all (for example, SB202190) reduce or inhibit the induction of ALP expression [44].

**Figure 7:**
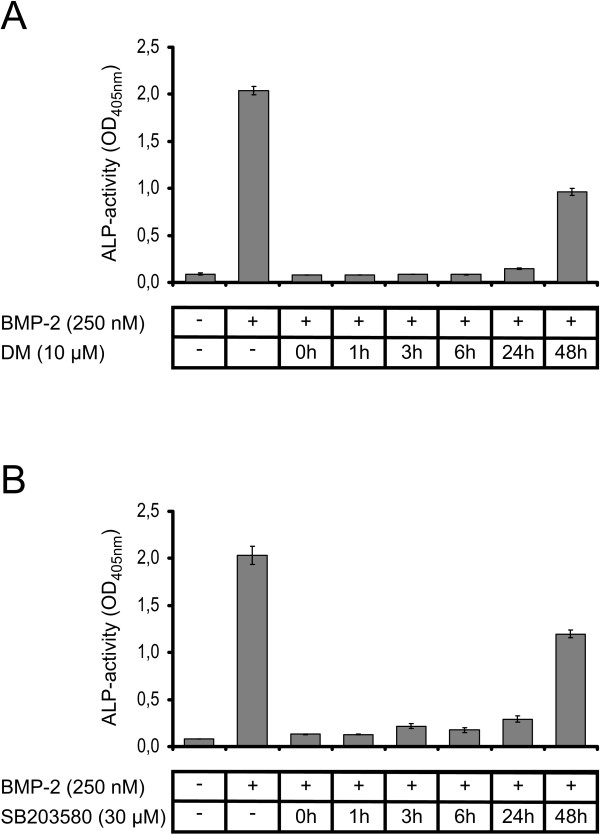
**Inhibition of the SMAD and p38 mitogen-activated protein (MAP) kinase pathway**. Alkaline phosphatase (ALP) assays were carried out using C2C12 cells in the absence or presence of 250 nM of bone morphogenic protein (wild type) (BMP2wt). **(a) **Dorsomorphin (DM) or **(b) **SB203580 was added at the indicated timepoints. The background absorption at 405 nm of 0.09 ± 0.0075 was not subtracted to indicate the signal to noise ratio.

To investigate if the activated SMAD1/5/8 and/or p38 MAP kinase pathways are required for the ALP induction, dorsomorphin and SB203580 were added separately to C2C12 cells at different timepoints using concentrations of 10 and 30 μM, respectively. ALP activity was analyzed 72 h after ligand addition (Figure 7). The results clearly show that the simultaneous administration of either dorsomorphin (Figure 7a) or SB203580 (Figure 7b) with ligand (that is, at t = 0 h) completely abolishes ALP induction. Even if the inhibitors are added 24 h after the ligand only a marginal increase in ALP activity is observed. Addition of the inhibitors 48 h after ligand administration still results in a significantly reduced ALP activity compared to induction by 250 nM BMP2wt in the absence of these inhibitors. Similar results were obtained from analogous experiments using ATDC-5 cells, thus the observed inhibition of ALP activity is not cell type specific (data not shown). These results clearly demonstrate that the induction of ALP gene expression requires a permanent activation of both, MAP kinase and SMAD pathways.

## Discussion

In this study we investigated the binding properties of different BMP ligands, *in vitro *and on whole cells, and correlated these properties with immediate downstream signaling events such as SMAD phosphorylation and induction of ALP expression. By performing *in vitro *interaction analyses in an identical manner for three prototypic members of the BMP subfamilies (BMP2/4, BMP5/6/7 and GDF5/6/7) we could compare and analyze differences in detail, enabling us to deduce consequences for the initial steps of receptor binding and activation. Due to the dimeric nature of the BMP/TGFβ ligands their receptor binding mechanism is inherently complex, which complicates data acquisition and analysis. The binding of the ligand to membrane-anchored receptors is affected by avidity as the two receptor sites lead to a strong increase in the apparent binding affinity. The increase in affinity should mainly result from slower dissociation because, based on statistical thermodynamics, it is highly unlikely that a molecule attached to two receptors can leave both sites simultaneously. If re-binding is fast the dissociation is slowed down dramatically. By contrast, statistical thermodynamics also predicts that binding of a dimeric ligand to membrane-anchored receptors will occur via a stepwise process, since the direct binding of ligand to two receptors simultaneously (with respect to the timing of the binding events) involves a trimolecular reaction, which is a very rare event. Thus, mechanistically, the ligands will most likely bind initially to a single receptor chain (unless the receptors exist as preformed dimers on the cell surface) and a second receptor chain will subsequently be recruited into this membrane-bound complex.

Our experimental setup utilizing immobilization of either the ligands or the receptors allows the determination of the rate constants for each of these association and dissociation steps. With immobilized ligands our *in vitro *interaction analysis delivers binding constants and kinetics for the so-called 1:1 interaction, where the receptors bind independently and no cooperativity is observed, due to the absence of allosteric mechanisms or direct contacts between the extracellular domains of the receptors. This setup provides parameters that likely resemble the situation when ligand first encounters the cell surface, thereby binding to a single receptor. Our results show that for the interaction of BMP2 and GDF5, with type I receptors, as well as the binding of activin A to the type II receptors ActR-II and ActR-IIB, the 1:1 interaction occurs with 30-fold to 100-fold lower affinity than the 1:2 interaction, under the conditions tested. Binding of BMP2 and GDF5 to the type II receptors is even more affected by avidity, showing a 100-fold to 1,000-fold increase in affinity when going from a 1:1 to a 1:2 interaction scheme. As expected the increased binding affinities in the 1:2 interactions result from reduced dissociation rates but an increase in the association rates was also observed in most cases. However, in the 1:1 interaction scheme, the dissociation rates for ligand:type II receptor complexes are much faster than those observed for the ligand:type I receptor interaction. The deduced half-lives for complexes of BMP2 and GDF5 bound to one type II receptor are, at most, on the order of very few seconds, whereas binding of these ligands to one of their type I receptors results in complexes with half-lives on the order of several minutes. Thus, assuming single isolated receptors for either subtype, BMP2 and GDF5 likely bind first to a type I receptor and subsequently recruit a second receptor (which possibly could be either subtype) into the membrane-anchored binary complex. The initial complex of a ligand bound to two membrane-anchored receptors likely stabilizes the complex by lowering the dissociation rate due to avidity such that the receptor recruitment can proceed without the intermediate complex falling apart.

BMP7 seems different when compared with BMP2 or GDF5 as the 1:1 interaction scheme does not reveal a clear high affinity receptor for BMP7. The type I receptor BMPR-IB and the type II receptor ActR-II exhibit almost identical affinities for BMP7. However, the dissociation rates again show that the BMP7:BMPR-IB complex has a fivefold longer half-life than the BMP7:ActR-II complex, making a sequential mechanism with binding of BMP7 first to BMPR-IB more likely. Most importantly, these hypotheses are only valid under the assumptions that no other components affect the complex lifetime and that the receptor usage of a ligand *in vivo *solely depends on the receptor affinity, that is, the receptor with the highest binding affinity is the receptor to be recruited by this ligand. However, it is known that signaling of BMP7 and BMP6 involves the type I receptor ActR-I [45], which binds with at least 30-fold lower affinity (in the 1:1 interaction scheme) than the other type I receptors BMPR-IA and BMPR-IB. Furthermore, coreceptors such as members of the DRAGON/RGM family or β-glycan can influence binding by enhancing recruitment to the membrane surface and, in the case of DRAGON, even influencing receptor specificity [46]. Aside from the coreceptors, heparin binding sites in some of the BMP ligands also change their membrane localization, possibly forming a ligand species that is not soluble as assumed but is rather, at least in part, localized to the membrane even in a receptor-unbound state.

To determine the receptor architecture present on cells *in vivo *and to correlate the observation with the *in vitro *binding affinities, we additionally performed ligand-binding assays to whole cells. Interestingly, if the ligand concentration is sufficiently high, the resulting binding curves suggest the presence of two different receptor species on cell surfaces. Using BMPR-IA transfected cells, high and low affinity binding sites whose binding affinities correlate with the respective 1:2 and 1:1 interaction *in vitro *could be identified for wild-type BMP2. We could estimate that about 10% of the overall receptor sites represent high affinity and about 90% the low affinity sites. By using a heterodimeric BMP2 variant with only one functional type I receptor epitope, we could confirm the presence and the number of these low affinity sites. Correlating these observations with our *in vitro *interaction analysis we suggest that about 10% of the type I receptors are present as preformed dimers, thereby binding BMPs with very high affinity, whereas the majority of the BMP type I receptors are present as isolated single binding sites on cells.

For the BMP type II receptors the picture is similar. Despite the exclusive detection of sites correlating in their ligand binding affinities to the 1:2 interaction scheme, the number of sites is much lower than expected from expression levels being comparable to those of BMPR-IA. We suggest that due to the low binding affinities for the 1:1 BMP2:ActR-IIB interaction (approximately 3 μM, see table 2), significantly high levels of ligand specifically bound to monomeric type II receptors cannot be achieved due to increased unspecific interactions and solubility limitation of the BMP ligands. These data nevertheless clearly show that the receptor architectures on cells are heterogeneous before ligand binding.

There are reports in the literature that preformed receptors and single receptors (that are oligomerized by the ligand) can address different signaling pathways, namely that preformed receptors lead to induction of the SMAD pathway, whereas ligand-induced receptor oligomerization leads to activation of the p38 MAP kinase cascade [47]. We investigated the consequences of binding to the two different receptor species by analyzing the induction of ALP expression and measuring SMAD phosphorylation. The concentration for half-maximal ALP expression correlates with the affinity determined for the 1:1 ligand:type I receptor interaction. Furthermore the heterodimeric BMP2 variant BMP2wt/L51P with only one functional type I receptor epitope exhibits nearly the same half maximal effective concentration (EC_50_), strongly suggesting that ALP induction is mediated through single type I receptor sites rather than through preformed type I receptor complexes. This differs from SMAD phosphorylation, which requires subnanomolar concentrations of BMP2. Thus the EC_50 _of early SMAD activation is close to the type I receptor affinity measured for the 1:2 interaction scheme *in vitro*. With increased incubation time, the EC_50 _values increase attaining values between those of the affinities for the 1:1 and 1:2 ligand:type I receptor interactions. These results strongly suggest that the low ligand concentrations required for SMAD phosphorylation during short-term BMP2 exposure is likely mediated by preformed type I receptor dimers, which can bind BMP2 with very high affinities in the subnanomolar range. During extended exposure these preformed receptor dimers seem to disappear, probably due to internalization, while monomeric receptor chains predominantly remain at the cell surface. These receptor monomers bind wtBMP2, as well as the heteromeric wt/L51P variant with only one intact type I receptor site, with a lower binding affinity, resulting in a lower EC_50 _value. A constitutive endocytosis via clathrin-coated pits, was reported for the type I receptor BMPR-IA and BMPR-II, and also for BMPR-II, via caveola-like internalization [48]. It is, however, unknown whether the internalized preformed receptor complexes reappear at the cell surface as complexes that would thus reconstitute high affinity sites, or if additional processes inhibit such reappearance and thereby keep the ligand sensitivity low. It also remains unclear, if other mechanisms such as autoregulatory feedback loops trigger, for example, total SMAD levels throughout the time the experiments take.

Our results utilizing BMP2 heterodimers with one ablated receptor epitope also clearly suggest that only one type I receptor is needed in the ligand:receptor complex to allow signaling. In contrast, earlier studies showed that two type II receptors are required for the formation of a signaling competent complex. It remains questionable whether this finding is the result of reduced type II receptor binding affinities (that is, thermodynamically controlled) or shorter half-lives of individual ternary complexes (that is, kinetically controlled). However, the recruitment of the type II receptors seems to be the limiting step in BMP2-mediated signaling.

The remaining single type I receptor sites are still capable of transducing signals via the SMAD pathway, but due to their lower ligand affinities higher concentrations are likely required. As shown by addition of dorsomorphin and SB203580, a sustained activation of the type I receptor resulting in both activated SMAD and MAP kinase pathways is required for the induction of BMP-responsive ALP gene expression. It is important to note that ALP is not directly activated by either SMAD and/or the p38 MAP kinase pathways, since cycloheximide abolishes BMP2 induced ALP mRNA synthesis [49]. It was demonstrated that BMP2 controls ALP expression and osteoblast mineralization by a Wnt autocrine loop. Consequently, BMP2-mediated ALP gene expression seems to depend only on the quantity of type I receptors being activated by the ligand. Subsequent processes seem not to be limiting.

## Conclusion

A comparison of our results obtained from *in vitro *interaction analyses with binding studies performed on intact cells provides new insights into the complexity of BMP/GDF receptor activation and its relevance for subsequent signaling events. Our data clearly demonstrate the presence of distinct receptor arrangements on the cell surface, contributing to distinct cellular responses. A minor subset of receptors seems to be preformed and contains at least two receptors of each subtype, allowing the assembly of active signaling complexes at low ligand concentrations. Whether this heterohexameric ligand:receptor arrangement acts as a functional unit or is part of even higher ordered cell surface structures remains undetermined. However, the majority of activated BMP receptors on cell surfaces mediating long-term signal transduction by BMP2 most likely consist of the ligand, two type II receptors but only one type I receptor chain. Such an assembly also best explains the signaling capabilities of heterodimeric ligands such as BMP2/7. If only one anchoring receptor can provide sufficient complex stability the recruitment of a variety of low affinity receptor chains into signaling complexes might be possible. Heterodimeric receptor arrangements (that is, ActR-I and BMPR-IA) were recently reported that were shown to be important for the signaling of homodimeric ligands such as BMP2 and BMP4 [50]. Thus, further analyses of ligand receptor interactions, and the identification of residues determining binding affinity and specificity of individual ligands to their receptors, might allow the construction of new homodimeric or heterodimeric ligand proteins with unique signaling capabilities. Moreover, the data presented here indicate a much greater complexity in receptor recruitment and activation, as well as in resulting downstream signaling events, than is typically appreciated for the BMP receptor system. The presumed discrepancy resulting from the disparity between ligand number and available receptor molecules, compared to highly specific biological functions addressed by the individual TGFβ family members, suggests that receptor complexes of identical composition but formed by different ligands can activate distinct signal cascades. This raises questions how parameters such as the order of receptor recruitment, complex lifetime, receptor stoichiometry, binding kinetics, and subtle differences in ligand receptor architectures can alter ligand specific signaling in quantity and/or quality. The data presented here provide a first glimpse of how some of the aforementioned parameters influence signaling by BMPs.

## Methods

### Expression and purification of receptor ECDs

The receptor ECDs of the type I receptors hActR-I (amino acids 21 to 123 [51]), hActR-IB (amino acids 24 to 126 [51]), hBMPR-IA (amino acids 24 to 152 [51]) and mBMPR-IB (amino acids 14 to 126 [52]), and of the type II receptors hActR-II (amino acids 18 to 135 [53]), mActR-IIB (amino acids 19 to 128 [54]), and hBMPR-II (amino acids 32 to 150 [13]) were expressed with a C-terminal thrombin cleavage site (LVPRGS) followed by a His_6_-tag in baculoviral infected S*f*9 insect cells as described previously [32]. After metal affinity chromatography (nickel-nitrilotriacetic acid (Ni-NTA); Qiagen, Hilden, Germany) the receptor proteins (BMPR-IA, BMPR-IB, and the type II receptor ECDs) were further purified by affinity chromatography using a BMP2 affinity resin as described [55]. Monomeric ECDs of Act-RI and ActR-IB were isolated by anion exchange and subsequent RP-HPLC. The receptor ectodomains of BMPR-IA, BMPR-IB, and ActR-IIB were additionally prepared as thioredoxin fusion proteins in *Escherichia coli *and purified as described previously [55].

### Expression and purification of ligands

The mature part of hBMP2 (amino acids 283 to 396 plus an N-terminal Met-Ala [55]) and GDF5 (amino acids 387 to 501 [56] plus a N-terminal Met-Lys) were expressed in *E*. *coli*, isolated from inclusion bodies, renatured and purified as described previously [25]. Recombinant h-activin A [57] with a His_6 _tag and a thrombin cleavage site located C-terminal of the furin cleavage site RXXR was expressed in S*f*9 insect cells. The non-glycosylated, biologically active protein was isolated from the conditioned medium by Ni-NTA chromatography, the His_6 _tag was removed by thrombin and the resulting protein was further purified by anion exchange chromatography and RP-HPLC. CHO-cell derived h-activin A and hBMP7 were purchased from R&D Systems, Minneapolis, MN, USA.

### Preparation of heteromeric BMP2 mutants

A sequence encoding for a thrombin cleavage site (TCS; amino acids: LVPRGS) was introduced at a position four amino acids N-terminal of the first conserved cysteine of BMP2. Mutations for the amino acid exchanges L51P [33] or A34D [32] were established by site directed mutagenesis. For the production of heterodimeric BMP2 mutants a BMP2 variant with an altered N-terminal sequence (MAPTSSSTKKTQLS) followed by a TCS site was prepared, which exhibits a lower pI due to a smaller number of positively charged site chains. The heterodimeric BMP proteins were produced and purified as described previously [40]. Homodimeric and heterodimeric BMP2 variants were enzymatically cleaved and the products purified by RP-HPLC.

### SPR measurements

A Biacore 2000 system (Biacore, GE Healthcare, Chalfont St. Giles, GB) was used for all biosensor experiments. Receptor ectodomains were *N*-biotinylated by incubation with equimolar concentrations of sulfo-NHS-LC-biotin (Pierce, Thermo Scientific, Rockford, IL, USA) as described previously [58]. Ligands were biotinylated using the same procedure but using a twofold molar excess of sulfo-NHS-LC-biotin. Using these conditions the majority of the molecules should statistically be biotinylated only at a single site leaving the majority of the binding epitopes unaffected. Proteins (approximately 200 resonance units (RU)) were immobilized to streptavidin-coated biosensor CM5 chips as described previously [25]. Interaction sensorgrams were recorded at a flow rate of 10 μl/min at 25°C. The association and dissociation times were set to 5 min. After each data acquisition cycle the biosensor chips were regenerated with 4 M MgCl_2 _for 2 min. The formation of ternary complexes was recorded as described previously [59]. Briefly, ligands at 100 nM in HBS500 buffer (10 mM HEPES, pH7.4, 500 mM NaCl, 3.4 mM ethylenediaminetetraacetic acid (EDTA), 0.005% surfactant P20) were first perfused for 2 min at a flow rate of 10 μl/min at 25°C over the biosensor surface coated with the high affinity receptor chain followed by a 2 min perfusion with 100 nM of the ligand plus 0 to 64 μM of soluble receptor ectodomain proteins. After a 5 min dissociation period, the chip was regenerated as described above.

### Evaluation of recorded sensorgrams

All apparent binding affinities were obtained using the software BIAevaluation v. 2.2.4 (Biacore, GE Healthcare, Chalfont St. Giles, GB). Affinities of 'slow' interactions characterized by low values for the association rate *k*_on _(<10^6 ^M^-1 ^s^-1^) and *k*_off _(<10^-2 ^s^-1^) were derived by fitting the kinetic data to a 1:1 Langmuir binding model (*K*_D _(kin)) since a dose-dependent equilibrium binding could not be achieved at low analyte concentrations. To exclude the effect of analyte rebinding, *k*_off _was evaluated only in the very early section of the dissociation phase. The association rate *k*_on _was fitted in a section for the association phase that shows a linear interdependence in the derivative ln(abs(dy/dx)). (abs: absolute value; y: resonance units (RU); x: time (s))

Since the data acquisition cannot be performed at sampling rates greater than 2.5 Hz, the fitting of 'fast' interactions with *k*_on _and *k*_off _values exceeding 10^6 ^M^-1 ^s^-1 ^(for *k*_on_) and 10^-2 ^s^-1 ^(for *k*_off_) is not applicable due to low amounts of data points in sections that are unbiased by mass transport limitation or analyte rebinding. In these cases the apparent binding affinities were determined from the dose dependency of equilibrium binding (*K*_D _(eq)). The relative standard deviations for mean *K*_D _(eq) values are below 25%. The evaluation of the dissociation rate *k*_off _and the association rate *k*_on _revealed relative standard deviations of <30% and <20%, respectively. Consequently, the relative standard deviation of *K*_D _(kin) is below 50%. Differences in binding affinities of more than a factor of two can therefore be considered as significant.

### Iodination of ligands

Ligand proteins were radiolabeled with ^125^I using the chloramine-T method as described previously [60]. All reactions were performed at room temperature using 100 pmol of ligand proteins and a twofold molar excess (200 pmol, 400 μCi) of Na^125^I (GE Healthcare, Chalfont St. Giles, GB) in a total volume of 20 μl. Labeled proteins were purified by gel filtration using a Sephadex P6DG column (Bio-Rad, Hercules, CA, USA). Typically, 50% to 60% of ^125^I is incorporated resulting in proteins containing statistically one label with a specific activity of approximately 2 μCi/pmol of ligand. The monoclonal anti-BMPR-IA Fab fragment (AbyD) was radiolabeled using 1,3,4,6-tetrachloro-3,6-diphenylglycoluril (iodogen) as described previously [61].

### Radioligand binding assays

Transfected COS-7 cells were seeded in Dulbecco's modified Eagle medium (DMEM) medium containing 10% fetal calf serum (FCS) into 24-well plates at a density of 7.5 × 10^4 ^cells per well. After 12 h of incubation at 37°C and 5% CO_2 _the cells were washed twice with serum-free DMEM medium. Then, 300 μl of incubation medium (DMEM without carbonate buffer, supplemented with 25 mM HEPES pH 7.5 and 0.1% bovine serum albumin (BSA)) was added and the cells chilled down to 4°C. A total of 300 μl of incubation medium containing radioactively labeled ligand proteins at varying concentrations was added and the cells were incubated for 3 h at 4°C. Unspecific binding was determined by adding a 1,000-fold molar excess of unlabeled ligand to the binding reaction. After incubation the cells were washed three times for 5 min at 4°C and incubated overnight at 4°C in 1 ml of lysis buffer (20 mM HEPES pH 7.4, 1% Triton X-100, 10% glycerol, 0.1% BSA). An aliquot (800 μl) of each lysate was analyzed using a γ counter.

### ALP assay

The promyoblast C2C12 cell line (ATCC CRL-172) was cultivated in DMEM containing 10% FCS, 100 U/ml penicillin G and 100 μg/ml streptomycin. ATDC-5 cells (RIKEN, Ibaraki, Japan; RCB0565) were grown in DMEM/Ham's F12 medium (1:1) containing the same antibiotics but 5% FCS. The ALP assays were carried out in 96-well microplates as described previously [25,32]. For inhibition of the SMAD and p38 MAP kinase pathway dorsomorphin (Merck, Darmstadt, Germany) or SB203580 (Calbiochem, Merck, Darmstadt, Germany) was added at the indicated timepoints.

### SMAD phosphorylation

SMAD phosphorylation was analyzed in C2C12 cells. Briefly, the cells were grown under serum starvation conditions and ligand proteins were added at different concentrations. At given timepoints the cells were lysed and 70 μg of the lysate were analyzed by SDS-PAGE. SMAD-1 and pSMAD-1 were detected by western blotting using a specific antibodies (Cell Signaling Technology, Danvers, MA, USA). The blots were quantitatively analyzed using the software ImageJ (National Institute of Health, Bethesda, MD, USA).

## Authors' contributions

KH performed binding studies on whole cells and SMAD phosphorylation assays. WSch performed mass spectrometical analysis and participated in ligand processing. AS performed ALP assays. TDM participated in ligand construction and purification. WS participated in BIAcore measurements and analyses. JN conceived the study and participated in all stages of the work. TDM and JN wrote the manuscript. All authors read and approved the final manuscript.

## Supplementary Material

Additional file 1 **Influence of ionic strength and pH on binding affinities**. Bar diagrams representing mean values of the binding affinities determined from Biacore measurements for the interaction of bone morphogenic protein (BMP)2 with the indicated immobilized receptor ectodomains (ECDs) at different ionic strength **(a) **and pH value **(b)**. The experiments were carried out in duplicate using six different ligand concentrations. Click here for file

Additional file 2 **Sample trace of individual ligand receptor interactions**. Binding of bone morphogenic protein (BMP)7 at a concentration of 80 nM to immobilized receptor ectodomains in absence or presence of 0.36% 3-[(3-cholamidopropyl)dimethylammonio]-1-propanesulfonate (CHAPS). Click here for file

Additional file 3 **Binding kinetics of ligand receptor interactions**. Examples of biosensorgrams of the interactions of bone morphogenic protein (BMP)2 with BMP receptor (BMPR)-IA and/or activin receptor (ActR)-IIB. At timepoint 0 the perfusion of the analyte with varying concentrations was initiated and stopped after 300 s **(a, b)**. Sections used for analyzing the kinetic rate constants *k*_off _and *k*_on _are indicated by arrows **(a1)**. The evaluation of the *K*_D _value from the dose dependence of equilibrium binding is shown as inset **(a2)**. Resonance units achieved in the binding equilibrium were plotted against the used analyte concentrations and fitted to the Michaelis-Menten equation yielding values for *R*_max _and *K*_D _(eq). A ternary complex was formed by perfusing immobilized BMPR-IA at timepoint 0 with 100 nM of BMP2 followed after 120 s by the perfusion with 100 nM of the ligand plus the indicated concentrations of ActR-IIB **(c)**. Sensorgrams revealed two types of binding kinetics: A 'slow' kinetic typical for the BMP2 type I receptor interaction **(a1, b1) **and a 'fast' kinetic typical for BMP2 type II receptor interactions **(a2, b2)**. Both kinetics can be observed during ternary complex formation **(c)**. Click here for file
